# Deep learning in CT colonography: differentiating premalignant from benign colorectal polyps

**DOI:** 10.1007/s00330-021-08532-2

**Published:** 2022-01-26

**Authors:** Philipp Wesp, Sergio Grosu, Anno Graser, Stefan Maurus, Christian Schulz, Thomas Knösel, Matthias P. Fabritius, Balthasar Schachtner, Benjamin M. Yeh, Clemens C. Cyran, Jens Ricke, Philipp M. Kazmierczak, Michael Ingrisch

**Affiliations:** 1 Department of Radiology, University Hospital, LMU Munich, Marchioninistraße 15, 81377 Munich, Germany; 2 Radiologie München, Burgstraße 7, 80331 Munich, Germany; 3 Department of Medicine II, University Hospital, LMU Munich, Marchioninistraße 15, 81377 Munich, Germany; 4 Department of Pathology, University Hospital, LMU Munich, Marchioninistraße 15, 81377 Munich, Germany; 5 Comprehensive Pneumology Center (CPC-M), Member of the German Center for Lung Research (DZL), Max-Lebsche-Platz 31, 81377 Munich, Germany; 6 Department of Radiology and Biomedical Imaging, University of California, San Francisco, 513 Parnassus Ave, San Francisco, CA 94117 USA

**Keywords:** Colonography, Computed tomographic, Colonic polyp, Deep learning, Early detection of cancer

## Abstract

**Objectives:**

To investigate the differentiation of premalignant from benign colorectal polyps detected by CT colonography using deep learning.

**Methods:**

In this retrospective analysis of an average risk colorectal cancer screening sample, polyps of all size categories and morphologies were manually segmented on supine and prone CT colonography images and classified as premalignant (adenoma) or benign (hyperplastic polyp or regular mucosa) according to histopathology. Two deep learning models SEG and noSEG were trained on 3D CT colonography image subvolumes to predict polyp class, and model SEG was additionally trained with polyp segmentation masks. Diagnostic performance was validated in an independent external multicentre test sample. Predictions were analysed with the visualisation technique Grad-CAM++.

**Results:**

The training set consisted of 107 colorectal polyps in 63 patients (mean age: 63 ± 8 years, 40 men) comprising 169 polyp segmentations. The external test set included 77 polyps in 59 patients comprising 118 polyp segmentations. Model SEG achieved a ROC-AUC of 0.83 and 80% sensitivity at 69% specificity for differentiating premalignant from benign polyps. Model noSEG yielded a ROC-AUC of 0.75, 80% sensitivity at 44% specificity, and an average Grad-CAM++ heatmap score of ≥ 0.25 in 90% of polyp tissue.

**Conclusions:**

In this proof-of-concept study, deep learning enabled the differentiation of premalignant from benign colorectal polyps detected with CT colonography and the visualisation of image regions important for predictions. The approach did not require polyp segmentation and thus has the potential to facilitate the identification of high-risk polyps as an automated second reader.

**Key Points:**

*• Non-invasive deep learning image analysis may differentiate premalignant from benign colorectal polyps found in CT colonography scans.*

*• Deep learning autonomously learned to focus on polyp tissue for predictions without the need for prior polyp segmentation by experts.*

*• Deep learning potentially improves the diagnostic accuracy of CT colonography in colorectal cancer screening by allowing for a more precise selection of patients who would benefit from endoscopic polypectomy, especially for patients with polyps of 6–9 mm size.*

**Supplementary Information:**

The online version contains supplementary material available at 10.1007/s00330-021-08532-2.

## Introduction

Colorectal cancer is one of the three most frequent cancer-related causes of death among men and women [1]. However, its mortality and incidence can be significantly decreased by early detection of precancerous adenomatous polyps which grow over several years [2–5]. Screening methods such as immunochemical faecal occult blood test and optical colonoscopy (OC) are proven to reduce mortality from colorectal cancer, particularly since clinical symptoms are often non-specific or absent [6, 7].

A non-invasive screening method for colorectal cancer is computed tomography (CT) colonography. For the detection of colorectal polyps ≥ 6 mm, the sensitivity of CT colonography is comparable to OC [8–10]. Computer-aided detection (CAD) algorithms can reduce the number of missed colorectal polyps at CT colonography when used as a second reader [11, 12].

However, conventional CT colonography does not allow a clear distinction between benign and premalignant colorectal polyps, which would be essential for individual risk stratification and therapy management. Premalignant adenomatous polyps require endoscopic resection, whereas benign findings of hyperplastic polyps avoid unnecessary interventions. As polyp size is the only surrogate indicator of the likelihood of malignancy at CT colonography, current guidelines recommend the resection of colorectal polyps ≥ 6 mm detected in CT colonography (European Society of Gastrointestinal and Abdominal Radiology, United States Multi-Society Task Force on Colorectal Cancer) [13, 14].

First studies have shown that machine learning–based CT colonography using radiomics may allow non-invasive differentiation of benign and premalignant colorectal polyps [15, 16]. These radiomics approaches consist of three steps. First, segmentation of the region-of-interest in the medical image, i.e. the polyp in the CT colonography scan. Second, extraction of radiomics features for the segmented regions. Third, machine learning analysis of the extracted features to predict polyp character. Especially the first step of polyp segmentation, which has been performed manually by experts, is a large barrier for the potential integration of these approaches into the clinical routine and prevents fully automated polyp classification. In addition, the interpretability of these approaches is limited to the importance of individual radiomics features. Deep learning could potentially overcome these challenges and thereby substantially reduce the gap to clinical applicability for machine learning–based polyp classification in CT colonography.

Deep learning–based image classification using convolutional neural networks (CNNs) does not require prior segmentation of the region-of-interest and has proven to be an efficient method in automated image analysis, providing a powerful tool for tumour detection and classification in oncologic imaging [17]. In the first step of a deep learning approach, a localisation of the polyp is sufficient. In the second step, a deep learning model can directly predict polyp character using a small subvolume of the CT colonography image around the localisation. Additionally, CNNs can be exploited to visualise regions in the input image that are potentially important for model predictions to achieve improved model interpretability [18].

Therefore, the aim of this study was to establish the differentiation of premalignant (i.e. adenoma) and benign (i.e. hyperplastic polyp or regular mucosa) colorectal polyps in CT colonography using deep learning.

## Materials and methods

### Training set

**Fig. 1:**
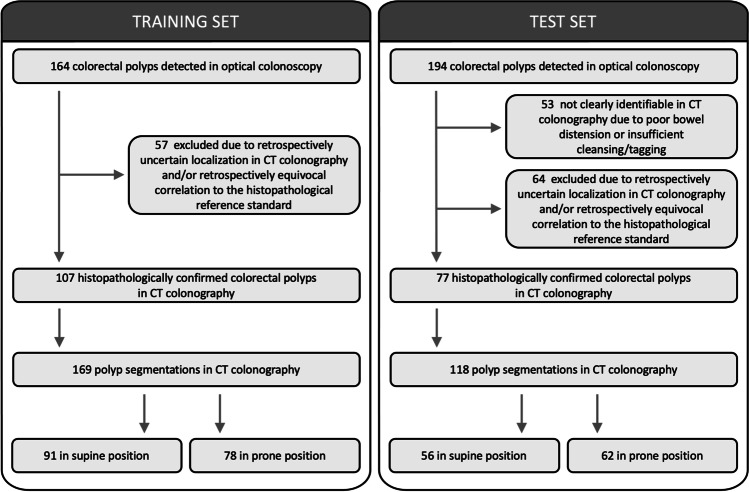
Flow diagram of the training set and the external test set

This study was approved by the institutional review board and the requirement for written informed consent was waived. It is a retrospective analysis of CT colonography images from a previously published prospective colorectal cancer screening cohort of an asymptomatic, average risk population over 50 years of age who underwent same-day OC and CT colonography [8]. Exclusion criteria of the previously published colorectal cancer screening cohort were signs of colonic illnesses such as abdominal pain, relevant changes in stool frequency, diarrhoea, melaenic stools, and haematochezia as well as positive family history for colorectal cancer, hereditary colorectal cancer syndromes, inflammatory bowel disease, severe cardiovascular or pulmonary disease, body weight > 150 kg, and prior OC within the last 5 years. Only participants with histopathologically confirmed findings corresponding to CT colonography findings were included in the present study (Fig. 1).

### CT colonography in the training set

CT colonography bowel preparation is described in the Supplemental Material. CT colonography images were acquired on a 64-channel multidetector row scanner (Siemens Somatom Sensation 64, Siemens Healthineers) at 0.6 mm collimation and reconstructed using a standard soft tissue kernel at a slice thickness of 0.75 mm and 0.5 mm reconstruction increment. Tube voltage was 120 kVp at tube current–time product reference values of 70 mAs in supine and 30 mAs in prone position using automatic tube current adaption. Mean radiation dose for CT colonography was 4.5 (0.6) mSv. For bowel distension, room air or CO_2_ was insufflated through a rectal tube. No intravenous contrast agent was administered. The CT colonography protocol was described in detail before [8].

### External test set

CT colonography datasets from a North American multicentre CT colonography screening trial, publicly available via The Cancer Imaging Archive (TCIA), served as an external test set [19–21]. The external test set comprised multicentre CT colonography images acquired on various CT scanners from different vendors (Siemens Healthineers; Philips Healthcare; GE Healthcare Systems; Canon Medical Systems) with varying scanning protocols. Polyps were only included if histopathologic reports were available.

### Polyp segmentation

**Fig. 2:**
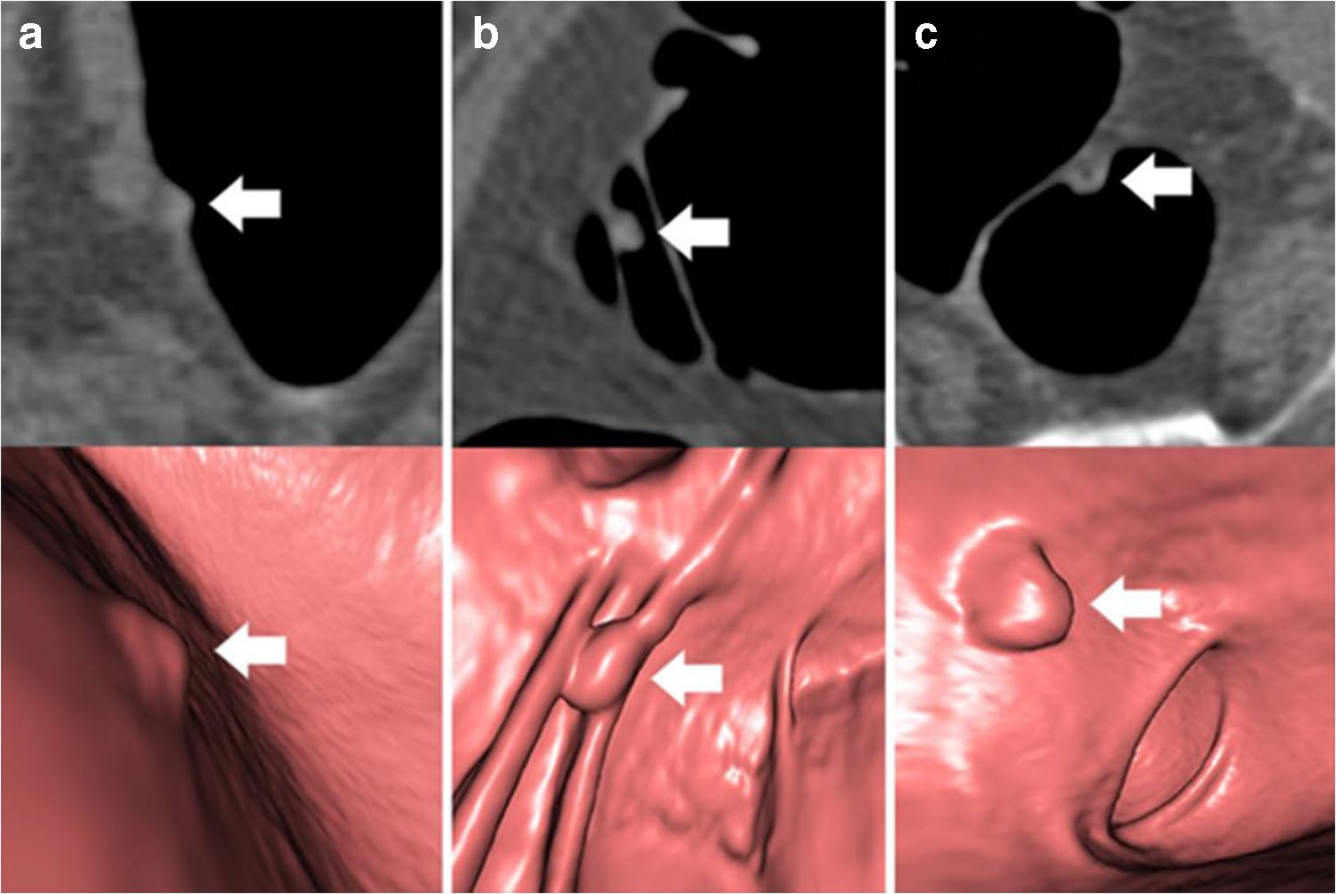
**a-c** Colorectal polyps of the training set (indicated by arrows) in axial 2D CT colonography images (top row) and in the corresponding virtual fly-through 3D reconstructions (bottom row). **a** 7-mm hyperplastic polyp in the rectum of a 58-year-old woman. **b** 8-mm tubular adenoma in the transverse colon of a 74-year-old woman. **c** 9-mm tubulovillous adenoma in the rectum of a 67-year-old man

Prospective polyp detection and polyp matching are described in the Supplemental Material. All readers were informed about polyp size and colon segment in which polypectomy was performed. Histopathological polyp class was blinded for all readers. Colorectal polyps were manually segmented in multiplanar 2D CT colonography images by a board-certified radiologist (8 years of experience in CT colonography imaging; completed a specialised hands-on workshop on CT colonography) and two radiology residents (3 years of experience in CT colonography imaging; one completed a specialised hands-on workshop on CT colonography) in equal amounts, as described in detail beforehand [16]. For exact retrospective polyp re-detection, 2D and virtual fly-through 3D CT colonography reconstructions were used (Fig. 2). Colorectal polyps that could not be clearly identified in CT colonography and/or unequivocally assigned to the corresponding histopathological report were excluded. A consensus reading was performed in case of divergent reading results. Consensus was reached when all three readers agreed on polyp localisation and segmentation. Each colorectal polyp was segmented in supine and prone position images, if confidently detectable in both positions. The CT colonography workflow of the dedicated post-processing software syngo.via versionVA30B (Siemens Healthineers) was used for polyp detection. The Medical Imaging Interaction Toolkit (MITK) Version 2018.04 (German Cancer Research Center — Division of Medical Image Computing) was used for polyp segmentation [22].

### Histopathological reference standard

A colorectal polyp was considered benign if the corresponding histopathological report classified it as “regular mucosa” or “hyperplastic polyp”, premalignant if the corresponding histopathological report classified it as “tubular adenoma”, “tubulovillous adenoma”, or “villous adenoma”.

Solely for study purposes, 2 lesions with the histopathological classification “serrated adenoma” and 3 lesions with the histopathological classification “adenocarcinoma” (39 mm, 44 mm, and 75 mm) were included in the group premalignant. One polyp with the histopathological classification “lipomatous” was included in the group benign (Table 1).

**Table 1:** Colorectal polyp segmentations in the training set and external test set class-divided according to the histopathological report

Histopathologic category	Number of polyp segmentations		Classification
	Training set	External test set	
Regular mucosa	3/169 (2%)	9/118 (8%)	Benign
Hyperplastic polyp	78/169 (46%)	30/118 (25%)	
Lipomatous polyp	2/169 (1%)	0/118 (0%)	
Tubular adenoma	57/169 (34%)	49/118 (42%)	Premalignant
Tubulovillous adenoma	16/169 (9%)	26/118 (22%)	
Villous adenoma	8/169 (5%)	0/118 (0%)	
Serrated adenoma	4/169 (2%)	0/118 (0%)	
Adenocarcinoma	1/169 (1%)	4/118 (3%)	

The adenocarcinoma segmentations were included in the premalignant group for study purposes only

### Deep learning–based ensemble models

**Fig. 3:**
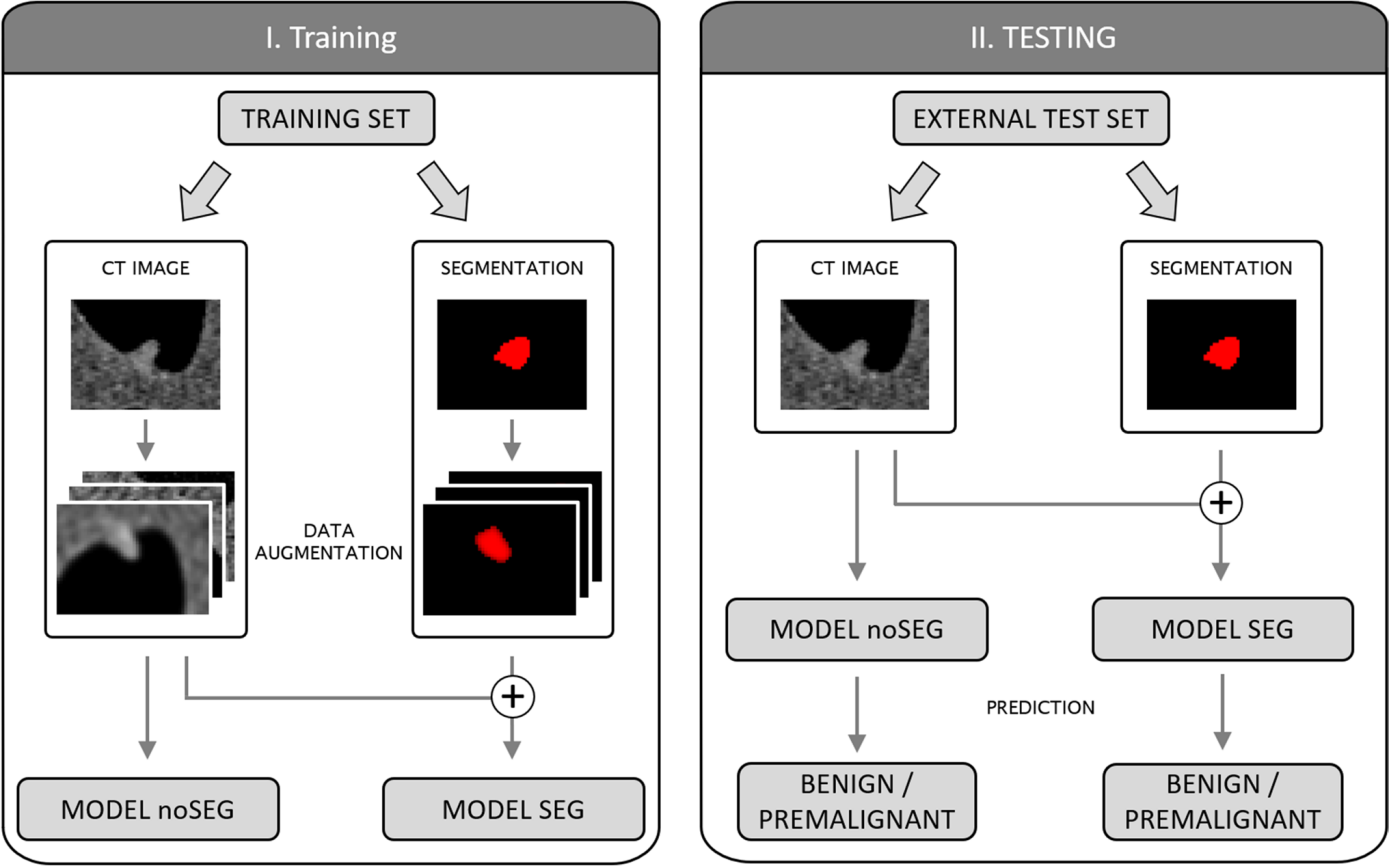
Schematic illustration of model training (left) and testing (right). Training: Model noSEG was trained on augmented CT images of the training set, model SEG was trained on augmented CT images and manual polyp segmentation masks. Testing: Model noSEG predicted polyp class (benign vs. premalignant) on CT images of the independent external test set, and model SEG made predictions based on CT images and manual polyp segmentation masks

**Fig. 4:**
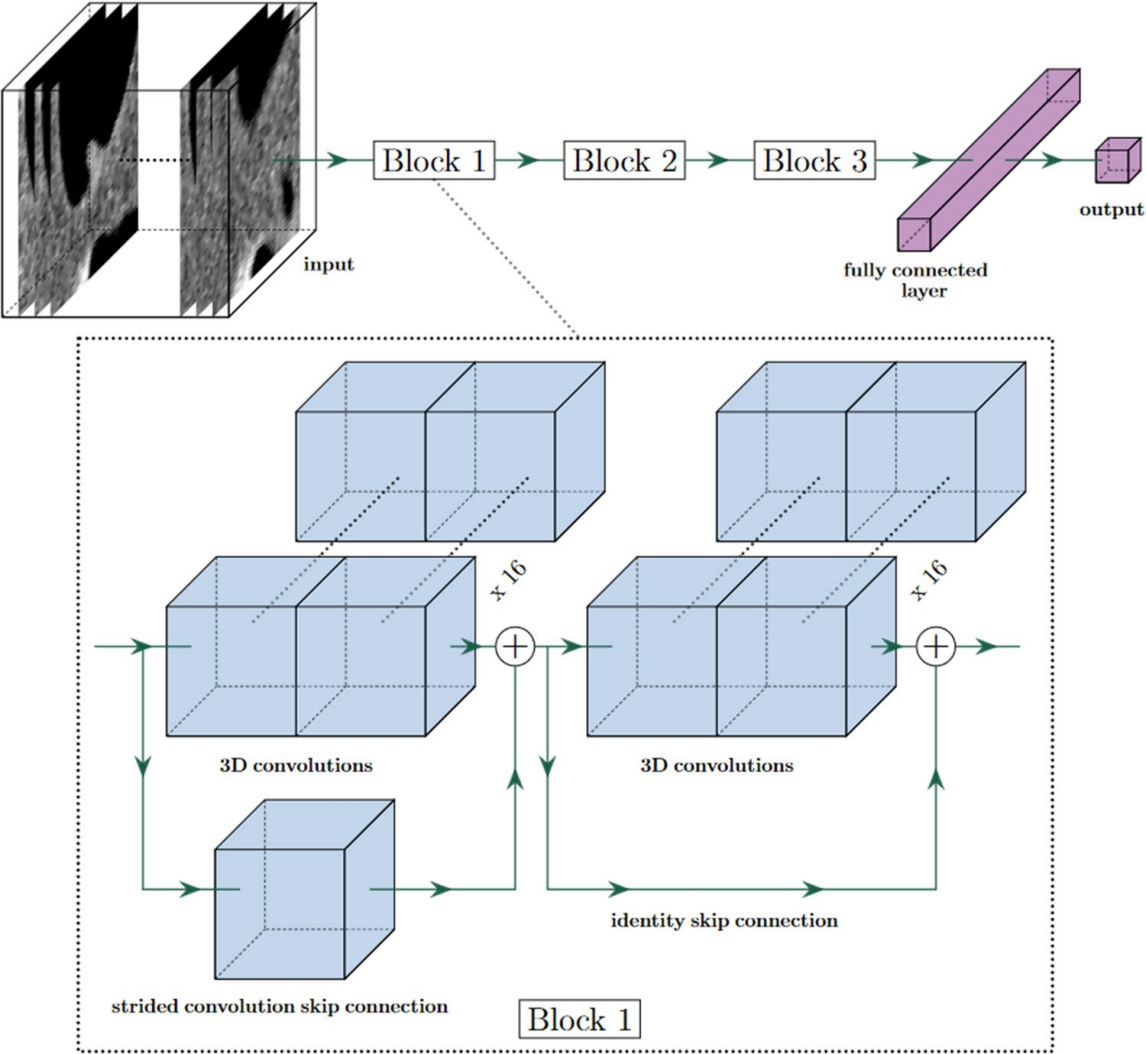
Schematic illustration of the CNN architecture used in the ensemble models SEG and noSEG. First, the input (CT image for model noSEG, CT image and manual polyp segmentation mask for model SEG) propagates through three convolution blocks (blocks 1, 2, and 3), each consisting of two consecutive three-dimensional convolutions with an increasing number of filter kernels (block 1: 16 kernels, block 2: 32 kernels, block 3: 64 kernels) and skip connections. Afterwards, a fully connected layer mapped the information to the output neuron which holds the output score (0.0 = benign, 1.0 = premalignant)

This study investigated two deep learning–based models, SEG and noSEG. Both models were ensembles, each consisting of 50 three-dimensional convolutional neural networks [23]. In each ensemble, the mean output of the 50 respective CNNs was used as model output. Ensembling was implemented to address the variance observed while training single CNNs. This variance was believed to be an effect of training set size — deep learning is typically applied on large datasets — and could not be eliminated with data augmentation. The CNNs used in both ensemble models were, apart from the input layer, identical (Fig. 3). CNNs in SEG expected inputs of size 50 × 50 × 50 × 2 (image + segmentation), CNNs in noSEG expected inputs of size 50 × 50 × 50 × 1 (image). A CNN from model noSEG is shown schematically in Fig. 4 and a detailed layer-by-layer description for CNNs from both models is provided in Table 2. Both models were implemented with Keras (version 2.4.3) [24], an open-source Python interface for neural networks. The open-source machine learning library TensorFlow (Google Brain, version 2.4.1) [25] was used as backend.

**Table 2:** Layer-by-layer description of the CNNs used in the two ensemble models SEG and noSEG

Name	Layer	Filter kernel (shape, count)		Output size	
		Main branch	Shortcut	noSEG	SEG
in	Input	-		50 × 50 × 50 × 1	50 × 50 × 50 × 2
res1a	3D convolution	3 × 3 × 3, 16	3 × 3 × 3, 1	25 × 25 × 25 × 16	
res1b	3D convolution	3 × 3 × 3, 16	id	25 × 25 × 25 × 16	
add1	Add	-		25 × 25 × 25 × 16	
res2a	3D convolution	3 × 3 × 3, 32	3 × 3 × 3, 1	13 × 13 × 13 × 32	
res2b	3D convolution	3 × 3 × 3, 32	id	13 × 13 × 13 × 32	
add2	Add	-		13 × 13 × 13 × 32	
res3a	3D convolution	3 × 3 × 3, 64	3 × 3 × 3, 1	7 × 7 × 7 × 64	
res3b	3D convolution	3 × 3 × 3, 64	id	7 × 7 × 7 × 64	
add3	Add	-		7 × 7 × 7 × 64	
pool	Global average pooling	-		64	
drop	Dropout	-		64	
out	Fully connected layer	-		1	

The convolutional part of each network (up to layer “add3”) consisted of a main branch, containing three-dimensional convolutions, and a shortcut branch, containing either a single convolution kernel for downscaling or an identity mapping (“id”). At each add layer (“add1”, “add2”, “add3”), the main branch and the shortcut branch were added. After add1 and add2, the images were split up again into main and shortcut branches

### CNN training

Every CNN in each of the models was trained individually to predict the histopathological polyp class label (benign vs. premalignant). CNNs in SEG were trained with images and segmentations; CNNs in noSEG were trained with images exclusively (Fig. 3). Every CNN was trained with a different 80–20 train-validation split. In these splits, 80% of the data were randomly selected as training data to train the network, and the other 20% were used as validation data to monitor the training process. Training parameters included a stochastic gradient descent (SGD) optimiser, a learning rate of 0.01, a binary cross-entropy loss function, and a batch size of 8. Data augmentation, including random cropping, was used in the training data. The validation data was not augmented, but cropped to size 50 × 50 × 50 around the polyp centre to match the input size. Early stopping was applied to automatically end the training process: If the AUC in the 20%-validation set did not increase for 64 epochs, training was stopped and the weights from the epoch with the highest validation AUC were restored.

### Statistical analysis of the external test set

The classification performance of the trained models SEG and noSEG was evaluated on the independent, external test set (Fig. 3). Model output scores were calculated as the arithmetic mean of the 50 individual output scores of the CNNs in each ensemble for each input image. The model output score was turned into a prediction using a classification threshold. The threshold was selected to yield a sensitivity of 80%. Classification performance was quantified using AUC, sensitivity, and specificity. For polyp size–based subgroup analyses, the maximum polyp diameter in three dimensions was calculated based on the polyp segmentation masks.

### Visual explanation of model predictions

**Fig. 5:**
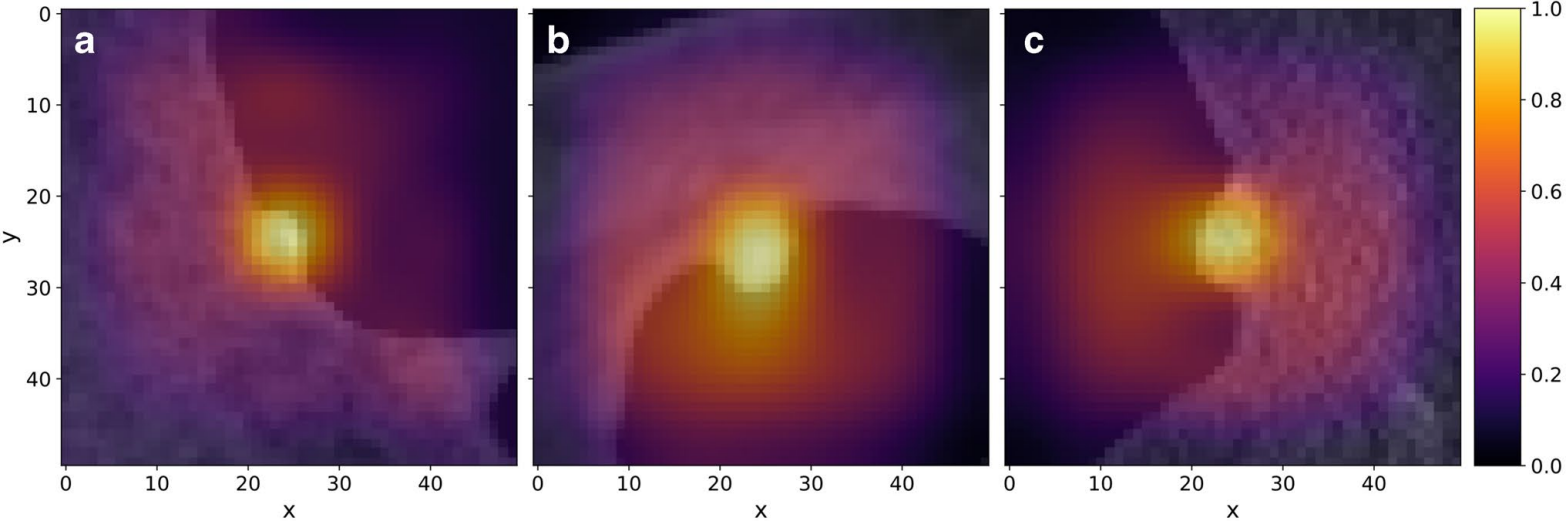
GradCAM++ images of model noSEG for the inputs (**a**) 7-mm hyperplastic polyp, (**b**) 7-mm tubular adenoma, and (**c**) 9-mm tubulovillous adenoma from the test set superimposed with the respective 2D CT colonography images. Grad-CAM+ + is a gradient-based explanation method for CNNs and was used to visualise the correspondence (0.0 = no correspondence, 1.0 = highest correspondence) of each image voxel with the prediction of the model noSEG (benign vs. premalignant polyp) [18]

The gradient-based CNN visualisation technique GradCAM++ [18] provided visual explanations of predictions made for the test set by model noSEG (predictions based on input images exclusively). For each voxel in an input image, GradCAM++ calculated a class activation, ranging from 0.0 to 1.0, to visualise the correspondence with the model output score. GradCAM++ images for three selected polyps are shown in Fig. 5. In addition, we quantified how much attention the model noSEG paid to voxels labelled as “polyp”, according to the manual polyp segmentation masks, during decision-making and calculated the percentage of voxels inside the manual polyp segmentation mask which had a GradCAM++ class activation of 0.25 or higher (Fig. 5).

The code for the statistical analysis was made publicly available on the development platform GitHub at https://github.com/pwesp/deep-learning-in-ct-colonography.

## Results

### Training set

Of 311 consecutively enrolled adults undergoing same-day CT colonography and OC, 2 had to be excluded due to withdrawal from the trial after CT colonography and 2 because of incomplete OC, as reported previously [8]. Of 307 colorectal cancer screening participants of an average risk asymptomatic screening population, 201 participants without findings of histopathologically confirmed polyps were excluded. Of 106 participants with histopathologically confirmed polyps, 43 were excluded due missing or incomplete CT colonography datasets. Of 164 colorectal polyps detected in OC, 57 were excluded due to retrospectively equivocal assignment to the histopathological reference standard and/or retrospectively uncertain localisation in CT colonography, as described in detail previously [16]. Thirty-five of 57 excluded polyps were benign, and 22 of 57 were premalignant. Consensus reading was performed in 5 of 107 polyps. In total, 107 colorectal polyps with histopathological reference were evaluated in 63 patients (23 female; mean age: 63 ± 8 years) comprising 169 polyp segmentations in CT colonography images (91 in supine position and 78 in prone position). Eighty-six polyp segmentations were categorised as premalignant (adenoma), of which 8 were ≤ 5 mm, 18 between 6 and 9 mm, and 60 ≥ 10 mm, measuring the maximum 3D diameter of polyp segmentations. Eighty-three polyp segmentations were categorised as benign (hyperplastic polyp or regular mucosa), of which 16 were ≤ 5 mm, 49 between 6 and 9 mm, and 18 ≥ 10 mm.

### External test set

Due to insufficient cleansing/tagging or poor bowel distension, 53 of 194 colorectal polyps detected in OC were not clearly identifiable in CT colonography. Sixty-four polyps were excluded due to retrospectively equivocal assignment to the histopathological reference standard and/or retrospectively uncertain localisation in CT colonography, as described in detail before [16]. Fifty-eight of 117 excluded polyps were benign, and 59 of 117 were premalignant. Consensus reading was performed in 5 of 77 polyps. In total, 77 colorectal polyps were analysed in 59 patients comprising 118 polyp segmentations (56 in supine position and 62 in prone position). Seventy-nine polyp segmentations were categorised as premalignant (adenoma), of which 1 was ≤ 5 mm, 30 between 6 and 9 mm, and 48 ≥ 10 mm. Thirty-nine polyp segmentations were categorised as benign (hyperplastic polyp or regular mucosa), of which 8 were ≤ 5 mm, 26 between 6 and 9 mm, and 5 ≥ 10 mm.

### Statistical analysis of the external test set

**Fig. 6:**
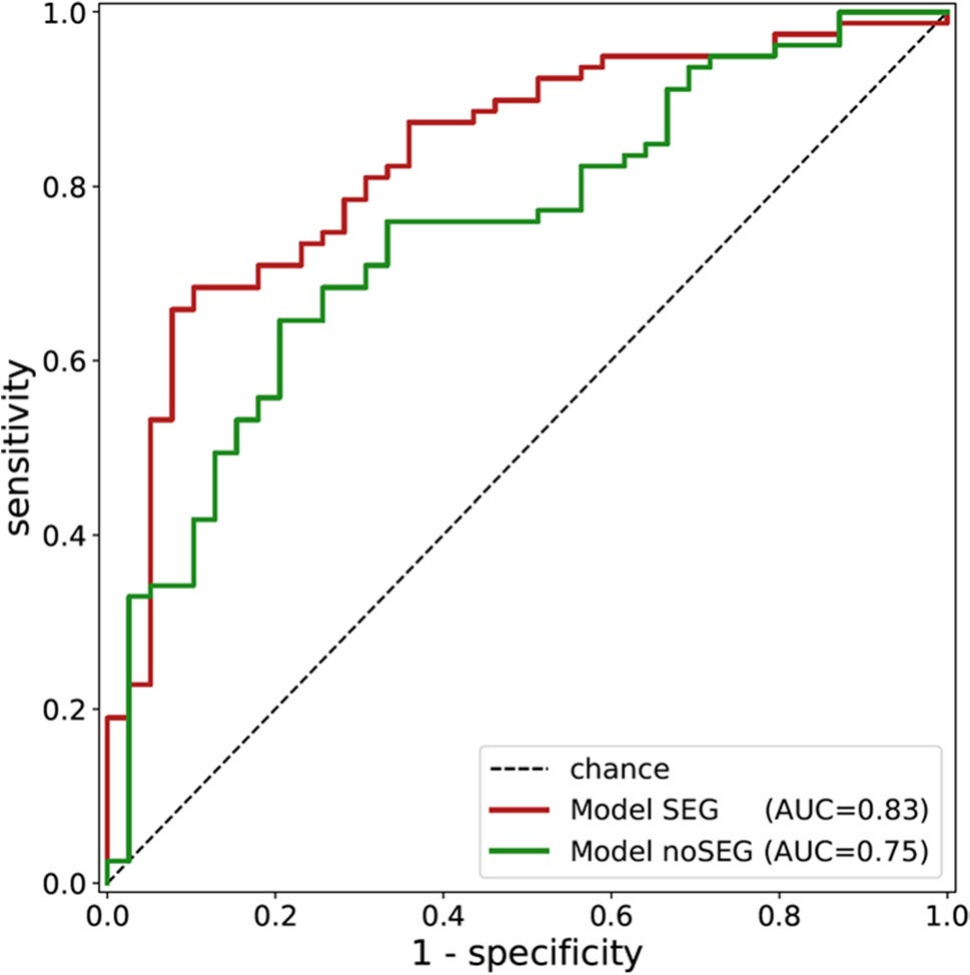
Receiver operating characteristic (ROC) curve for deep learning predictions of polyp class (benign vs. premalignant) in the external test set from model SEG and model noSEG

On the independent, external test set, output scores from model SEG yielded an AUC of 0.83, and output scores from model noSEG yielded an AUC of 0.75. Model predictions for polyp class from model SEG yielded a sensitivity and specificity of 80% (63 of 79) and 69% (27 of 39) for a classification threshold of 0.27. noSEG predictions for polyp class yielded a sensitivity and specificity of 80% (63 of 79) and 44% (17 of 39) for a classification threshold of 0.36 (Fig. 6).

Visual explanations of deep learning predictions were provided using the gradient-based CNN visualisation technique GradCAM++. The fraction of manual polyp segmentation mask voxels which had a GradCAM++ class activation of 0.25 or higher from model noSEG was 90% on average.

In size-based subgroup analyses of the external test set, model SEG yielded an AUC of 0.74 for polyps with a size between 6 and 9 mm and 0.72 for polyps ≥ 10 mm. Model noSEG yielded an AUC of 0.72 for polyps with a size between 6 and 9 mm and 0.74 for polyps ≥ 10 mm. As current guidelines recommend endoscopic resection for colorectal polyps ≥ 6 mm, the number of polyps ≤ 5 mm with available histopathologic classification in the external test set (9 polyp segmentations) was not sufficient to provide reliable results for this size category [13, 14].

In a further subgroup analysis of the external test set based on the histopathologic report (see Table 3), tubulovillous adenoma had the highest percentage of correctly classified cases (SEG: 23/26 (88%); noSEG: 23/26 (88%)), followed by tubular adenoma (SEG: 36/49 (73%); noSEG: 37/49 (76%)) and hyperplastic polyp (SEG: 21/30 (70%); noSEG: 15/30 (50%)).

**Table 3:** Class prediction accuracy of the two models SEG and noSEG on the external test set polyp segmentations for each histopathologic category

Histopathologic category	Model accuracy		Ground truth classification
	SEG	noSEG	
Regular mucosa	3/9 (33%)	7/9 (78%)	Benign
Hyperplastic polyp	21/30 (70%)	15/30 (50%)	
Lipomatous polyp	0/0	0/0	
Tubular adenoma	36/49 (73%)	37/49 (76%)	Premalignant
Tubulovillous adenoma	23/26 (88%)	23/26 (88%)	
Villous adenoma	0/0	0/0	
Serrated adenoma	0/0	0/0	
Adenocarcinoma	4/4 (100%)	3/4 (75%)	

The four adenocarcinoma segmentations were included in the premalignant group for study purposes only

## Discussion

In this proof-of-concept study, we investigated the deep learning–based differentiation of premalignant and benign colorectal polyps in CT colonography datasets of an average-risk, asymptomatic colorectal cancer screening cohort of over 50 years of age. Deep learning–based image analysis allowed for the differentiation of benign and premalignant colorectal polyps with CT colonography with an AUC of 0.83. Even when manual polyp segmentations were not used for decision-making, deep learning reached an AUC of 0.75. External validation demonstrated robustness of the deep learning models, despite images acquired with heterogeneous CT colonography imaging protocols on various CT scanners [19–21]. Tubulovillous adenomas were classified with higher accuracy (88% each model) compared to less premalignant tubular adenoma (73% and 76%). This might indicate that, for premalignant polyps, the differentiation performance is increased with higher malignant potential of polyps.

The use of deep learning for the classification of colorectal polyps in CT colonography is not yet well established. In a pioneering study, Tan et al. investigated a deep learning–based classification of colorectal lesions > 30 mm detected in CT colonography in correlation to the histopathological reference standard [26]. Tubular adenoma, tubulovillous adenoma, and villous adenoma were labelled as benign (*N* = 31); adenocarcinomas were labelled as malignant (*N* = 32). In two-fold cross validation, a deep learning model trained on CT colonography images reached an AUC of 0.84 [26].

Our study adds to the literature, as we showed the ability of deep learning–based image classification at CT colonography to differentiate between adenomas (premalignant) and hyperplastic polyps (benign), considering that most colorectal cancers develop from adenomas and the incidence of colorectal cancer can be significantly decreased by early detection with subsequent resection [2–4]. As we included polyps ≤ 9 mm (*N* = 91 images in the training set, *N* = 65 images in the external validation), our results show that small colorectal polyps can be classified as benign or premalignant using deep learning. Furthermore, we evaluated the performance of our deep learning–based models in an independent, external, multicentre test set.

Besides deep learning, classical machine learning methods have been used for colorectal polyp classification in CT colonography as part of a radiomics approach [15, 16]. Radiomics approaches typically consist of three steps: region-of-interest segmentation, radiomics feature extraction, machine learning prediction. In a previous analysis of this training and external test dataset using such a radiomics approach, a random forest machine learning model enabled the robust differentiation of benign and premalignant CT-colonography-detected colorectal polyps with an AUC of 0.91 [16]. The higher performance compared to deep learning (AUC of 0.84 and 0.75) can be attributed to the relatively small size of the training dataset. Deep learning typically requires larger amounts of data for successful training than classical machine learning methods like random forests [17, 27].

The present study provides additional value as, contrary to a radiomics approach, deep learning–based CT colonography image analysis did not require polyp segmentation. Merely a localisation of the polyp had to be provided. Additionally, deep learning models extract image features and make predictions at the same time, which leads to an approach with just two steps: localisation and deep learning prediction. This promises application in clinical routine, since polyp localisation would be more feasible compared to a thorough segmentation. Furthermore, it provides the basis for a fully automated CT colonography evaluation as the deep learning–based polyp classification could be combined with already established CAD algorithms for polyp detection [11, 12]. Additionally, the CNNs which made up the deep learning models enabled the visual interpretation of predictions. We used the gradient-based CNN visualisation technique GradCAM++ [18] to highlight regions in the input CT colonography image that were potentially relevant for decision-making. High activation in image regions that were manually labelled by radiologists to create polyp segmentation masks confirmed that model noSEG was capable of recognising autonomously which image voxels were important for decision-making, without the need for pre-identification via polyp segmentation. In contrast, radiomics approaches typically allow to rank features according to their importance during training a classical machine learning model. However, the majority of radiomics features are second-order texture features which are difficult to interpret in a medical context.

Used as a second reader, deep learning–based CT colonography analysis could further increase the clinical impact of CT colonography–based colorectal cancer screening by enabling a more precise selection of patients who would profit from subsequent endoscopic polypectomy. Particularly considering that colorectal cancer screening programs using CT colonography showed higher participation rates compared to OC [28, 29]. Current guidelines recommend the resection of colorectal polyps ≥ 6 mm detected in CT colonography [13, 14]. One reason for this recommendation is that colonoscopic referral for polyps with a size of ≤ 5 mm at screening CT colonography has been shown to have very poor cost-effectiveness with $464,407 per life-year gained [30]. Furthermore, Pickhardt et al. demonstrated that the incremental cost-effectiveness ratio of colonoscopic referral for polyps with a size between 6 and 9 mm at CT colonography was $59,015 per life-year gained, compared to − $151 (cost savings per person) for polyps with a size of ≥ 10 mm [30]. By allowing the differentiation of premalignant from benign colorectal polyps, especially in the size category between 6 and 9 mm, deep learning–based CT colonography analysis could potentially increase the cost-effectiveness ratio of colonoscopic referral after CT colonography.

This study has limitations. The sample size was small. Every polyp securely identifiable in CT colonography and unequivocally assignable to the corresponding histopathological report was segmented. A substantial number of polyps detected in OC, however, had to be excluded. Therefore, the results of this study are only applicable to polyps clearly detectable in CT colonography and a selection bias cannot be fully ruled out. No polyp that was presented to a deep learning model during training was presented to the model again during testing. However, correlations within multiple segmentations of one polyp or within multiple polyps of one patient in model SEG cannot be ruled out. The prevalence of serrated adenomas in this study (1.6%) (2 out of 122 patients) was in agreement with the prevalence of serrated adenomas in a large-scale CT colonography screening study (1.4%) [31]. However, the number of serrated adenomas (*N* = 2) was not sufficient to provide reliable results for deep learning–based analysis of this category.

## Conclusions

In this proof-of-concept study, deep learning–based analysis of CT colonography allowed differentiating premalignant from benign colorectal polyps in an external validation cohort corresponding to histopathology. Differentiation was possible, even when the model was provided only CT images and did not utilise expert polyp segmentation masks. Deep learning allowed for visual interpretability of the results so that image regions potentially important for predictions could be analysed. Although the findings need to be validated in prospective studies, the presented method promises to facilitate the identification of high-risk polyps as an automated second reader.

## Supplementary Information

Below is the link to the electronic supplementary material.

Supplementary file1 (DOCX 21 KB)

## Abbreviations

AUC: Area under the curve
CNN: Convolutional neural network
MITK: Medical Imaging Interaction Toolkit
OC: Optical colonoscopy
PEG: Polyethylene glycol solution
ROC: Receiver operating characteristics
TCIA: The Cancer Imaging Archive

## Declarations

### Guarantor

The scientific guarantor of this publication is Michael Ingrisch.

### Statistics and biometry

The authors Philipp Wesp, Balthasar Schachtner, and Michael Ingrisch have significant statistical expertise.

### Study subjects or cohorts overlap

Some study subjects or cohorts have been previously reported in Grosu S, Wesp P, Graser A, et al (2021) Machine Learning-based Differentiation of Benign and Premalignant Colorectal Polyps Detected with CT Colonography in an Asymptomatic Screening Population: A Proof-of-Concept Study. Radiology 202363. https://doi.org/10.1148/radiol.2021202363.

### Methodology

• retrospective

• experimental

• performed at one institution
