# Supplementary material for: Deep learning in CT colonography: differentiating premalignant from benign colorectal polyps
Source: Eur Radiol. 2022 Jan 26;32(7):4749–59. doi: 10.1007/s00330-021-08532-2 (PMC9213389; doi:10.1007/s00330-021-08532-2)
Supplement: Supplementary file 1 — Supplementary file1 (DOCX 21 KB) [file 330_2021_8532_MOESM1_ESM.docx]

**Supplement**

*CT colonography bowel preparation*

CT colonography bowel preparation included 4 L of polyethylene glycol solution (PEG) (KleanPrep, Norgine Pharmaceuticals and 20 mg bisacodyl with 30 mL of sodium phosphate (Prepacol, Guerbet Pharma). For faecal tagging 50 mL of iopamidol were added to the last litre of PEG (Solutrast 300, BraccoAltana Pharma). For bowel relaxation 20 mg n-butyl scopolamine (Buscopan, Boeringer Ingelheim Pharmaceuticals) were injected. No intravenous contrast agent was administered. Bowel preparation was described in detail before [1].

Polyp detection and matching

In the previously published colorectal cancer screening cohort, which was used as the training set, prospective colorectal polyp detection in CT colonography was performed by three board-certified radiologist who had read >300 CT colonography examinations prior to the study [1]. A polyp was rated as a true positive detection if it was localized in the same or adjacent colonic segment in CT colonography and OC, and if the measured size of the polyp was within the same size category or if there was a deviation of no more than one size category, as described in detail before [1].

In the CT colonography data sets available via TCIA, which was used as the test set, prospective colorectal polyp detection in CT colonography was performed by 15 radiologists who had read >500 CT colonography examinations prior to the study, or had participated in a specialized training on CT colonography, and had the highest scores on the qualifying examination, as described in detail before [2,3,4]. A polyp was rated as a true positive detection if it was localized in the same colonic segment in CT colonography and OC, and if the measured size of the polyp was within 50% of its reference standard measure derived from pathology (or OC in case of piecemeal or fulgurated resection), as described in detail before [2,3,4].

The information thus obtained on polyp size and localisation was used for retrospective polyp re-detection and polyp segmentation in the present study. Only true positive polyp detections were included in the present study.

*Image preprocessing and data augmentation*

Both CT colonography data sets, the training set and the external validation set, were preprocessed by cropping, thresholding and normalization before they were passed on to classification models. Images and polyp segmentation masks were cropped to a size of 100x100x100 voxels around the polyp center position, which was calculated using the segmentation. Voxel values in the image were cropped, such that values below -400 or above 400 Hounsfield units (HU) were set to -400 or 400 HU, respectively. During normalization, voxel values in the image were linearly rescaled using the min-max normalization $x' = (x + 400) / 800$, which mapped all voxel values from their original HU scale into the value range between 0.0 and 1.0 while preserving the physical attenuation coefficient information. Data augmentation was applied to cropped images and segmentations during training to enrich the training data with slightly modified images and segmentations in order to facilitate training deep networks. The following augmenting operations were deployed: random cropping (random selection of a 50x50x50 sub-image of the 100x100x100 image which included the entire polyp), elastic deformation, three-dimensional rotation, gamma correction, flipping along image axes, brightness transformation, adding of gaussian noise and gaussian blurring. For all data augmentation tasks we used the open-source python framework batchgenerators (Division of Medical Image Computing at the German Cancer Research Center, version 0.21) [5].

*CNN architecture*

The network design was deduced from the residual blocks of the ResNet-18 architecture [6] and used skip connections. Skip connections provided an extra path for the information to propagate through, bypassing the convolutional operations of the main path. This bypass addressed the “vanishing gradient problem” [7,8] and allowed training deeper networks. Network depth, i.e. the number of layers, is a crucial parameter in feed forward networks [7] and network performance was increased by increasing the number of layers in the past [8,9].

**References (Supplement)**

1. Graser A, Stieber P, Nagel D, et al (2009) Comparison of CT colonography, colonoscopy, sigmoidoscopy and faecal occult blood tests for the detection of advanced adenoma in an average risk population. Gut 58:241–248. https://doi.org/10.1136/gut.2008.156448
2. Smith K, Clark K, Bennett W, et al (2015) Data From CT_COLONOGRAPHY. Cancer Imaging Arch. https://doi.org/10.7937/K9/TCIA.2015.NWTESAY1
3. Johnson CD, Chen MH, Toledano AY, et al (2008) Accuracy of CT colonography for detection of large adenomas and cancers. N Engl J Med 359:1207–17. https://doi.org/10.1056/NEJMoa0800996
4. Clark K, Vendt B, Smith K, et al (2013) The Cancer Imaging Archive (TCIA): maintaining and operating a public information repository. J Digit Imaging 26:1045–57. https://doi.org/10.1007/s10278-013-9622-7
5. Isensee, Fabian, Jäger, Paul, Wasserthal, Jakob, et al (2020) batchgenerators - a python framework for data augmentation. Zenodo. https://doi.org/10.5281/zenodo.3632566
6. He K, Zhang X, Ren S, Sun J (2016) Deep Residual Learning for Image Recognition. In: 2016 IEEE Conference on Computer Vision and Pattern Recognition (CVPR). IEEE, Las Vegas, NV, USA, pp 770–778
7. Eldan R, Shamir O (2016) The Power of Depth for Feedforward Neural Networks. ArXiv151203965 Cs Stat
8. Szegedy C, Wei Liu, Yangqing Jia, et al (2015) Going deeper with convolutions. In: 2015 IEEE Conference on Computer Vision and Pattern Recognition (CVPR). IEEE, Boston, MA, USA, pp 1–9
9. Simonyan K, Zisserman A (2015) Very Deep Convolutional Networks for Large-Scale Image Recognition. ArXiv14091556 Cs
